# Social Perceptions of Nurses for Nursing Students

**DOI:** 10.1590/0034-7167-2025-0336

**Published:** 2026-07-24

**Authors:** Stéfany Araújo Fraga, Felipe Kaezer dos Santos, Luiza Mara Correia, Antonio Marcos Tosoli Gomes, Denize Cristina de Oliveira, Livia Fajin de Mello, Frances Valéria Costa e Silva, Helena Ferraz Gomes

**Affiliations:** I Universidade do Estado do Rio de Janeiro. Rio de Janeiro, Rio de Janeiro, Brazil

**Keywords:** Nursing, Social Representation, Nurses, Students, Nursing, Nursing Care., Enfermería, Representación Social, Enfermeros, Estudiantes de Enfermería, Atención de Enfermería.

## Abstract

**Objectives:**

to analyze the social representation of nurses among nursing students.

**Methods:**

a qualitative study, based on the structural approach of the Social Representation Theory. Participants were 114 nursing students from a public university in Rio de Janeiro. Data collection used the free word association technique, analyzed with the support of the EVOC^®^ software.

**Results:**

the likely central core of the representation was composed of the terms “care”, “empathy”, and “health”. The prominent peripheral elements were “love”, “knowledge”, “devaluation”, and “tiredness”. The contrast zone presented the terms “responsibility”, “assistance”, and “dedication”, reinforcing the terms of the central core.

**Final Considerations:**

the social representation of nurses is strongly structured around care, linked to empathy, knowledge, and ethical responsibility. Although permeated by affection and commitment, images related to devaluation and overload emerged, revealing tensions between the idealized training and the reality of the profession.

## RESUMEN

**Objetivos:**

analizar la representación social de las enfermeras entre estudiantes de enfermería.

**Métodos:**

estudio cualitativo basado en el enfoque estructural de la Teoría de las Representaciones Sociales. Participaron 114 estudiantes de enfermería de una universidad pública de Río de Janeiro. La recolección de datos se realizó mediante la técnica de asociación libre de palabras, analizada con el software EVOC^®^.

**Resultados:**

el probable núcleo central de la representación estuvo compuesto por los términos “cuidado”, “empatía” y “salud”. Los elementos periféricos prominentes fueron “amor”, “conocimiento”, “desvalorización” y “cansancio”. La zona de contraste presentó los términos “responsabilidad”, “asistencia” y “dedicación”, reforzando los términos del núcleo central.

**Consideraciones Finales:**

la representación social de las enfermeras está fuertemente estructurada en torno al cuidado, vinculada a la empatía, el conocimiento y la responsabilidad ética. Si bien está impregnada de afecto y compromiso, surgieron imágenes relacionadas con la devaluación y la sobrecarga, revelando tensiones entre la formación idealizada y la realidad de la profesión.

## INTRODUCTION

The concept of care is an intrinsic attribute of humanity and essential for the maintenance of humanity, being considered the essence of nursing. Thus, since the emergence of professional nursing, care has undergone very significant transformations, from the earliest, still quite primitive approaches to the consolidation of practices through evidence^(1)^.

With the evolution of care, nursing also progressed, especially from the 19^th^ century onwards, when Florence Nightingale cared for wounded soldiers in the Crimean War (1853-1856), applying basic concepts such as ventilation, hygiene, and nutrition to those wounded in combat^(2)^.

It is possible to assume that, at that time, the actions proposed by Florence Nightingale were based on her conception of nursing, differing from the prevailing understanding of nursing work up to that point, and driven by a unique and innovative conception of what it meant to be a nurse. Beyond direct patient care, it involved applying logical reasoning, meticulous observation, environmental organization, and ethical responsibility based on evidence. Based on scientific knowledge and her own practices, she proposed a model of care focused on health promotion and disease prevention, not just cure^(3)^.

In Brazil, nursing only came to be recognized as a science in the 20^th^ century, following the consolidation of theoretical models of care. Among these, the model proposed by Wanda de Aguiar Horta, focused on basic human needs and comprehensive care for individuals, stands out^(4)^. Over the years, various theoretical conceptions of care have emerged, contributing to the consolidation of nursing science based on the human being, the environment, and professional action^(5)^.

Amidst various advancements, there is currently a considerable challenge to overcome regarding social recognition of nursing. To this end, there are significant obstacles to overcome, such as precarious working conditions, devaluation due to gender issues, exhausting work hours, and low pay^(6)^. Overcoming these challenges is crucial to ensuring that nursing continues to play its transformative role in healthcare, strengthening public policies and academic spaces for training and research.

It must be considered that nurses’ performance and professional achievement are related to individuals’ perception of their professional category. Just as with Florence Nightingale, to occupy a certain position in society and the world of work, it is necessary for professionals to understand their role, responsibilities, competencies, and potential^(7)^.

In this regard, the training process takes central importance, since, throughout their undergraduate studies, nursing students advance in competencies and in the consolidation of their professional identity^(8)^. Therefore, understanding students’ perception of nursing can provide insights for reflecting on the recognition of the category and also on the influence of experiences arising from the formative process on this image.

Social representation is understood simultaneously in both individual and social terms. Individually, it encompasses subjects, referring to their thoughts. Socially, it is embedded within a group. Throughout life, thinking and representation can acquire new forms and possibilities^(9)^. According to Serge Moscovici, social representation consists of grouping images and language, focusing on situations constructed through social interactions, associating the compatibility of the thinking of groups and individuals. Therefore, representations contribute to the construction of what is generated externally according to the relationship that groups and individuals build with objects^(10)^.

A profession’s stereotypical representation has a significant impact on the perception of the work of that category. Historically, the image of nurses has been linked to a social representation based on religious values and charity. Although this perception has evolved over time, it still does not achieve full recognition of nursing^(11)^. Hence, it becomes important to identify nurses’ social representation.

Currently, the rapid circulation of information and the media’s strong influence significantly impact the social construction of nursing. The media, by disseminating stereotypes that portray nurses as professionals lacking autonomy, subordinate, and with limited knowledge, reinforces misconceptions that undermine their professional value^(12)^.

A study conducted in 2018 with approximately 60 nursing students showed that students’ social representations of nurses are still marked by traditional conceptions, often associated with the figure of altruistic caregivers and with care practices focused on charity and task execution. These perceptions influence the professional identity construction, risking compromising the appreciation of nurses’ social inclusion, autonomy, and recognition^(13)^.

In another study, nursing students reported frustration when confronting the ideal professional profile constructed in academia with practical reality, marked by professionals who are frequently exhausted and overburdened with multiple tasks. Furthermore, the study highlighted the imposition of care planning centered on medical interventions, generating conflicts and uncertainties about the identity and meaning of nurses’ professional practice in daily life^(14)^.

Therefore, it is necessary to consider that nurses’ social representation is a fundamental element in understanding how nursing is perceived by the professionals themselves in training. Such representations directly influence the professional identity construction, as well as the social and symbolic value of nursing. Unfortunately, it is common to observe the reproduction of stereotypes that associate nurses with the figure of an assistant or subordinate to doctors, reducing the complexity and autonomy that characterize nursing professional practice^(15)^.

Studying nurses’ social representation among nursing students is therefore relevant to identifying how these perceptions are formed and reproduced throughout their academic training. This becomes even more necessary in a context marked by overwork, multiple responsibilities, and lack of recognition-factors that demotivate nursing practice, directly impacting the quality of care offered^(16)^.

The relevance of this research lies in understanding how nurses’ representation is formed and transformed throughout their academic career, influencing their professional identity. This understanding highlights nursing as a profession in constant development and redefinition, reinforcing the importance of promoting reflections that value nursing, deconstruct stigmas, and reaffirm its essential role in healthcare.

## OBJECTIVES

To analyze nurses’ social representation among nursing students.

## METHODS

### Ethical aspects

The study followed CNS Resolution 466/2012^(17)^, which defines ethical guidelines for research with human beings, ensuring participants’ rights, safety and well-being. After institutional authorization, it was registered on *Plataforma Brasil* and approved by the Research Ethics Committee (Opinion 6,913,915). The inclusion of participants occurred through agreement with the Informed Consent Form, made available virtually according to Circular Letter 1/2021-CONEP/SECNS/MoH^(18)^. The document, in digital format, allowed for full reading, with electronic registration of acceptance and subsequent sending of a copy to participants.

### Theoretical-methodological framework

This study is based on the Social Representation Theory (SRT) theoretical and methodological framework. According to Serge Moscovici, social representations are complex phenomena that act directly on groups, being constructed through social interaction^(10)^. According to the author, there is a direct relationship between the individual and the collective. Thus, the notion of representation allows us to understand this link and how the constructed meanings can be shared^(19,20)^.

In order to achieve the objective proposed for this study, SRT’s structural approach was used, which has as a key element the central core theory, developed by Jean-Claude Abric in 1976^(21)^. In the structural approach, social representation is understood as an organized structure traversed by different dimensions. From this perspective, simply identifying the representation content is not enough; it is also necessary to understand how this content is structured and articulated internally^(22)^.

Therefore, the structural approach considers representation as an organized and structured set of information, beliefs, opinions, and attitudes, constituting a particular sociocognitive system composed of two subsystems: a central system (or core) and a peripheral system^(23)^. The structural approach utilizes prototypical analysis, which consists of organizing freely evoked words based on the mention of an inducing term, according to frequency and average order of evocation (AOE), and arranging them in a chart with four quadrants. This composition, in turn, presents the evoked terms in groupings that reveal the representation’s probable central core and its peripheral components^(24)^.

The choice of the structural approach was due to its widespread use and applicability, allowing for constructive dialogue with various areas of knowledge^(25)^. It is worth mentioning that the conception of this approach values an experimental methodological orientation, thus emphasizing the structuring of representations’ cognitive content^(26)^. Therefore, applying this approach allows us to broaden our understanding of an object of representation -nurse - for a social group: nursing students.

### Study design

This is a qualitative and descriptive study, structured based on COnsolidated criteria for REporting Qualitative research methodological principles, which offers standardized guidelines for presenting qualitative research, ensuring greater transparency, consistency, and rigor in the description of methodological procedures.

### Methodological procedures

Data collection took place between July 2024 and March 2025 using an electronic form organized into two sections: the first was for constructing the study group sociodemographic characterization; and the second was for collecting free word associations. Participants were asked to register up to five words for the inducing “nurse” term. Most studies using prototypical analysis generally request the association of three to five terms per participant, although this technique can also be used with a different number of responses and even without a restriction on quantity^(25)^.

### Study setting

The study setting was the nursing school of a public university in the state of Rio de Janeiro, which has 320 students distributed across nine academic semesters. Nursing students actively enrolled at the university were included, regardless of sexual orientation. Participants under 18 years of age or with prior training, at any level, in the health field were excluded. This decision was made to minimize the influence of prior experiences in the context of healthcare on data production.

### Data source

Students from two first-year classes and two from the ninth (and final) academic year were included. Thus, we had participants from different stages of the course, representing the beginning and end of their studies. This aimed to ensure heterogeneity in the sample, allowing for the capture of possible variations in social representations throughout the academic journey. The number of participants was determined by class selection, with voluntary student participation, ensuring an adequate sample size for identifying the representation’s central core and peripheral elements.

In total, 130 students responded to the data collection instrument, which is equivalent to 40.6% of the student body. Participant selection was carried out by convenience, a strategy widely used in qualitative research^(27)^, as it facilitates access to the study group and, in this case, allows the inclusion of students at different stages of the course.

### Data collection, organization, and analysis

Data collection was carried out by the study’s lead author, who at the time was working as a nursing intern at the institution where the research was conducted and with which she maintained a direct link. To carry out the data collection, the researcher received specific training from her supervising professor, based on reading and discussion.

Participants were approached in the classroom, with a detailed explanation of research objectives and procedures, and invited to participate voluntarily. Data collection occurred through a digital form, collectively but individually and without communication among participants. This strategy allowed for standardization of the instrument’s application and ensured that everyone correctly understood the instructions. This moment occurred only with the researcher and participants, without professors or other students.

The data produced by participants was organized in a spreadsheet and standardized into a dictionary of evocations. On average, four terms per participant were recorded, totaling 561 evocations, consolidated into 86 standardized terms. The analysis *corpus* was then submitted to EVOC^®^ software (version 2005), which distributed the words into the four quadrants of the four-quadrant method according to frequency and AOE.

Additionally, a similarity analysis was performed, a technique that studies the distances between different elements. The linking strengths of the terms are presented in tree form, showing the distances among them. Elements that are close together or connected to many others tend to be considered central. Conversely, those that are further away, with few links and low linking strength tend to be considered peripheral^(21)^.

**Figure 1:**
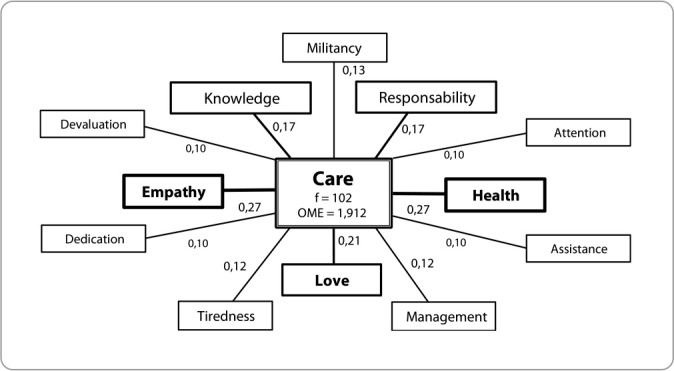
Maximum similarity tree (nurses’ social representation for nursing students), Rio de Janeiro, Rio de Janeiro, Brazil, 2025

Thus, a maximum similarity tree (Figure 1) was obtained from determination of co-occurrence between two evoked terms: the higher the frequency of co-occurrence, the greater the link strength. This index was calculated by the ratio between the number of co-occurrences of two terms in the analysis *corpus* and the number of participants. The maximum similarity tree construction was carried out by the authors of this study, prioritizing the connections with the greatest link strength.

## RESULTS

In Table 1, we present the research participant sociodemographic data to understand the profile of the studied group. The 114 participants are mostly female, totaling 94 participants (82.5%). The most predominant age groups were 18 to 20 years, with 41 participants (36%), and 21 to 23 years, with 37 participants (32.5%). Among participants, 64 identified as white (56.1%), and 30 participants (26.3%) identified as black.

**Table 1:** Participant sociodemographic characterization, Rio de Janeiro, Rio de Janeiro, Brazil, 2025

Variables	Nursing students									
	Man	%	Woman	%	No info.	%	Trans M.	%	TOTAL	%
18 to 20 years old	6	5.3	33	28.9	2	1.8	0	0.0	41	36.0
21 to 23 years old	4	3.5	32	28.1	1	0.9	0	0.0	37	32.5
24 to 26 years old	4	3.5	22	19.3	0	0.0	1	0.9	27	23.7
27 to 29 years old	2	1.8	2	1.8	0	0.0	0	0.0	4	3.5
30 years old or more	0	0.0	5	4.4	0	0.0	0	0.0	5	4.4
White	6	5.3	57	50.0	1	0.9	0	0.0	64	56.1
Black	6	5.3	23	20.2	1	0.9	0	0.0	30	26.3
Brown	4	3.5	14	12.3	1	0.9	1	0.9	20	17.5
TOTAL	16	14.0	94	82.5	3	2.6	1	0.9	114	100

*No inf. - not informed; Trans m. - transgender man.*

The analysis of the profile of the 114 participants puts into perspective the results of nurses’ social representation. The female predominance may reinforce symbolic dimensions associated with care, historically valued in nursing. Age range indicates the beginning of the group’s professional trajectory, which may influence the prioritization of more general or idealized elements of nursing. Furthermore, racial diversity, with a white majority and a significant presence of black students, may also affect the perception of professional experiences and values, reflecting in the central and peripheral organization of the social representation. Thus, gender, age, and life experience provide a relevant interpretative context for data analysis.

The four-quadrant method (Chart 1) is formed by terms evoked by participants, considering frequency and AOE. In the upper left quadrant, we have the central core, composed of stable elements. In other words, the cognemes in this quadrant are the representation’s consistent core, less affected by individuals’ external environment or activities. Thus, composing the probable central core of this study, we have the “care”, “empathy” and “health” elements. The “care” term was the most frequent (102) and the most quickly evoked (AOE of 1,912).

**Chart 1:** Four-quadrant method, Rio de Janeiro, Rio de Janeiro, Brazil, 2025

	AOE < 3.0			AOE < 3.0		
Freq. >= 22	Care Empathy Health	Freq. 102 27 25	AOE 1.912 2.963 2.480	Love Knowledge	Freq. 26 22	AOE 3.231 3.318
Freq. >= 10 < 21	Responsibility Assistance Dedication Welcoming	Freq. 18 16 16 10	AOE 2.444 2.313 2.875 2.700	Devaluation Fatigue Management Activism Humanization Work Assistance Attention	Freq. 19 16 15 14 12 12 11 11	AOE 3.526 3.625 3.200 3.143 3.583 3.083 3.091 3.455

*AOE - average order of evocation; Freq. - frequency.*

Subsequently, we have the representation’s peripheral elements, which provide the interface between concrete reality and the central system, carrying the group’s heterogeneity and apparent contradictions. The first periphery includes the most important elements, which, in certain situations, may prove to be central. The second periphery is composed of more volatile and changeable elements, depending on the most immediate experiences and influences on the group.

Thus, in the upper right quadrant, we have the representation’s first periphery, with a high frequency of evocation, but announced later. In this quadrant are the “love” and “knowledge” terms, with the former being the most frequently evoked and the most readily recalled. In the lower right quadrant, we have the second periphery, which includes words with a low frequency of evocation and pronounced later. The “devaluation” term stands out, with the highest frequency of evocation, although the most readily recalled term was “work” (AOE of 3.083). In the contrast zone, we have elements that are evoked quickly, but with a low frequency of evocation. In this region, there are terms such as “responsibility”, “assistance”, “dedication”, and “welcoming”.

Starting with the four-quadrant method, we present the maximum similarity tree (Figure 1), highlighting the connections between the elements that make up nurses’ representation for the studied group.

## DISCUSSION

The central core is composed of the most stable and consensual elements of the representation, which are highly shared by the group^(26)^. In this study, the likely central core presents three elements with distinct dimensions, namely “care” (conceptual), “empathy” (positive affective-attitudinal), and “health” (technical-professional). Thus, the central core takes on the features of a typically affective professional, marked by images that seem to indicate an idealized representation. This may be due to the fact that the studied group is composed of young nursing students, without prior experience in nursing or in the health field, which is evidenced by the absence of elements that express context, technique, and professional practice.

The “care” term is a multidimensional, broad, and considerably abstract conceptual element that, by itself, does not reveal the epistemological, ontological, and methodological components related to nurses’ social representation^(28)^. Hence, for the group studied, care is possibly part of the work process and is a primary function of nursing, encompassing a wide variety of activities. This care process involves other associated issues. Since it is a concept, it can include representations linked to psychological, physical, technical, and procedural aspects^(29)^.

When considering the set of elements that make up the likely central core, we observe that care is accompanied by a positive affective element (empathy) and an element associated with technical work for health production. This seems to suggest that care, as represented by study participants, may contain psycho-affective and relational elements, but also technical and practical components, resulting from the very tasks of caregiving. In this sense, it is worth reinforcing that caregiving in health involves theoretical, technical, and also scientific competencies, since it is possible that participants represent nursing as a science that has caregiving as an art, being present in all stages of life^(30)^.

The “care” term appears most frequently and is least frequently mentioned, indicating that it is the organizing element in the representation of the nurse. This shows that care is the fundamental value attributed to nursing, being perceived as essential. The presence of “empathy” and “health” in this quadrant reinforces the humanized image focused on others’ well-being, revealing a positive image strongly associated with the caregiving profile of nursing.

A study of social representations of “being a nurse” among nursing students also pointed to care as a central element, with similar behavior regarding frequency (f = 308) and AOE (1.960). According to the authors, despite the technical and scientific advancements in nursing, there is a natural tendency among nursing professionals to perform their duties in a more affective way, associating the more subjective aspects of nursing with the interaction with clients during their workday^(31)^.

This line of thought leads us to the first periphery of this study, which contains elements with high frequency but later evocation, indicating content more adaptable to subjects’ context and practical experience. In this area, the “love” term, with a positive affective-attitudinal character, was the most pronounced, remaining in this quadrant only due to its AOE.

The representation of love seems to refer to the origin of nursing, when the task of caring was performed by family members or people of religious charity as a way to atone for their faults. The love element is reinforced by the image of “dedication”, present in the contrasting area. Both terms indicate the persistent religious influence on nurses’ representation, established by the historical context of nursing’s creation, reinforcing the conception that this is the professional whose work demands selflessness and devotion^(32)^. The idealization of nursing, permeated by affection as an instrument of care and as an act of philanthropy, is associated with the image of professionals who do it out of vocation, out of the gift of caring for people, because, in principle, they are good and dedicated.

The “love” and “knowledge” terms, which comprise the first periphery of this study, are the same terms found in a previously mentioned study^(31)^. Knowledge, in contrast to the idea of care based on love, presupposes scientific grounding as a guiding principle of nursing. Appropriation of knowledge, in turn, seems to reinforce the “responsibility” term, also present in the contrasting area, insofar as responsible work is done with consistent theoretical bases.

Thus, while “love” indicates the affective and vocational component frequently attributed to nursing, “knowledge” refers to the scientific and technical dimension of nursing. The presence of these terms suggests an overlap between affective idealization and technical valuation, showing that nursing is considered not only as care permeated by compassion, but also as a scientifically grounded practice.

The second periphery, located in the lower right quadrant, presents the less frequent elements and those evoked later, reflecting contextual aspects and individual experiences. Here, components appear that are beginning to be part of nursing students’ professional lives, such as terms related to work, more specifically management, work, attention, and assistance, with the last two seeming to reinforce the sense of care already mentioned.

Furthermore, it presents two other negative affective-attitudinal elements in relation to the work of nurses, which are “devaluation” and “exhaustion”, expressing a more critical perception of professional reality, possibly influenced by internship experiences or by knowledge of reports about the precariousness of work. The devaluation of nurses is a discussion not only for the studied group, but a general idea of society about nursing. The lack of information about nurses’ role emphasizes and potentiates devaluation of nursing^(33)^.

Nursing students in Brazil often perceive nursing as undervalued, influenced by statements of adverse conditions during their training, such as excessive working hours, low wages, and rigid hierarchies. Other conditions, such as subordinate roles in clinical placements, lack of autonomy, and limited professional recognition, contribute to feelings of insecurity, low self-esteem, and doubts about their career choice. These feelings are reinforced by observing already graduated professionals who face exhausting work schedules, scarce institutional appreciation, and low pay^(34)^.

This perception has a significant impact, since student demotivation tends to increase dropout rates and reduce their engagement with nursing. Furthermore, a negative view of the career’s value can also limit the development of leadership skills and professional identity, as students begin to perceive nursing as a path of sacrifice with little return^(35)^.

Given the difficulties faced by the group, activism can be a defining element in the pursuit of better working conditions, recognition, and professional appreciation. The search for improved working conditions is often linked to the movements of the categories involved, since in Brazil the nursing workforce is predominantly composed of nursing assistants and technicians with secondary-level training who perform routine care tasks such as taking vital signs, administering medication, and providing hygiene care, often without a clear distinction between their duties^(36)^. Nurses, on the other hand, with higher education, take over roles of planning, coordination and clinical decision-making. This hierarchical division, on the one hand, expands access to healthcare, but, on the other hand, highlights wage and autonomy disparities^(37)^, as well as overlapping tasks on the part of technicians and assistants, resulting in fragility in professional practice^(36)^.

This reality has significant repercussions. Mid-level professionals frequently face precarious working conditions, job insecurity, and emotional overload, while the distinction among categories can generate conflict and moral distress within teams. Furthermore, wage inequalities are exacerbated by ethnic and racial issues, with white professionals receiving, on average, more than black and brown professionals. These factors compromise quality of care, reduce the visibility and recognition of nursing as an autonomous profession, demanding policies that value all categories and promote a clear definition of responsibilities and fair working conditions to strengthen the healthcare system^(38)^.

The elements located in the contrast zone have a low frequency, although they are evoked early. This configuration may indicate the existence of a subgroup of participants who attribute high relevance to certain image elements or, alternatively, simply reinforce the contents already present in the other zones of the four-quadrant method^(19,26)^, as we examine below.

Thus, we considered that the “assistance” term accompanies the sense of care and work, just as the “dedication” element can be associated with empathy, love, and humanization in the face of commitment with the other. The “responsibility” term points to a representation that highlights the seriousness of nurses’ work, while “assistance” and “welcoming” reinforce the relational and affective character of care. These elements may indicate a more committed and reflective view of nursing among some of participants.

It is important to highlight that the “welcoming” term, being eminently polysemous, carries different meanings depending on the context in which it is applied. In its most affective and relational sense, welcoming represents an act of empathetic listening, guided by respect for the other’s uniqueness, and is essential to the humanized practice of nursing. Thus, welcoming should favor the construction of bonds of trust, constituting the soft technology of care and guaranteeing the recognition of individuals as leading figures of their own care^(39)^.

On the other hand, welcoming takes on a structural dimension in healthcare services, especially in Primary Care and emergency services, considering triage or risk stratification. In this case, the initial listening and assessment of urgency are organized by protocols that guarantee access and technical prioritization, which gives an operational and institutional character to the term^(40)^.

Therefore, although the contrast zone elements could theoretically indicate the existence of representational subgroups, in the present study, this hypothesis was not confirmed. The analysis revealed that no element showed a differentiated distribution among student subgroups, and all terms in the contrast zone were connected to the central core elements, without forming differentiated groupings. Thus, the contrast zone seems to reinforce elements already present in the central core, confirming its complementary function in nurses’ representation.

Similarity analysis reinforces the central position of “care”, the element around which all others orbit. This data seems to suggest that nurses’ representations and of care overlap, due to the proximity between being and doing in nursing. Maximum similarity tree allows us to return to the discussion about the meanings of care, since, considering the linking forces among the terms evoked, all other elements are connected to it. Thus, given its relevance in nurses’ social representation, all other terms refer to “care”.

The “care” element brings together all the major linking forces, remaining at the center of the similarity tree, and the other terms, such as “empathy” and “health”, are strongly linked to it. We can also identify that the “responsibility” term, originally in the contrast zone, is much closer to centrality compared to other peripheral elements. This data is also similar to another study carried out with nursing students^(31)^, which has care and responsibility as components of the central core.

### Study limitations

Despite the relevance of the findings, the study is limited by the number of participants, the fact that it was conducted at only one higher education institution, and the lack of further tests to confirm the representation central elements. We wish to expand the number of participants and institutions, as well as explore the social representation of care in future studies, given its importance in this research and as a way to better understand nurses’ own representation, considering the connection between the two.

### Contributions to nursing, health, or public policy

At the end of the study, the findings allow us to reflect on the need to reconsider professional training strategies so that future nurses recognize and value themselves for the relevance of nursing and the quality of their work. Furthermore, we foresee the need to institute policies that promote better working conditions, career opportunities, and emotional support during training, strengthening the perception that nursing is an essential profession in the context of dignified and autonomous healthcare.

## FINAL CONSIDERATIONS

The findings of this study offer contributions to the field of nursing education, especially by highlighting how students construct and share social representations about nursing during their undergraduate studies.

In summary, nurses’ social representation is structured around care, anchored in values such as empathy and knowledge, based on a dual valuation: firstly, affection and commitment; secondly, technical and scientific competence. However, negative or critical representations linked to devaluation and work overload have also emerged, revealing the tension between the ideal of training and the reality of nursing.

The centrality of “care” confirms that the essence of nursing work is internalized from an early age as a commitment to others’ well-being. The way care is represented - permeated by affective values such as empathy, love, and dedication, and strained by elements such as devaluation and exhaustion - reveals an important duality that should be discussed in training settings. The “empathy” and “health” terms compose a humanized and holistically oriented image, highlighting the relational character that permeates nurses’ professional identity.

The presence of elements such as “love” and “knowledge” in the first periphery demonstrates the coexistence of affective and technical values, respectively, indicating a representation that recognizes both the emotional and scientific dimensions of professional practice. On the other hand, the emergence of terms such as “devaluation” and “exhaustion” points to a symbolic conflict between the formative ideal and the reality experienced or observed in the practical context, revealing the impacts of precarious work and the low social valuation of nursing.

These results reflect the existence of a social representation marked by erosion, in which both the symbolic valuation of caregiving and critical perceptions of the conditions of professional practice coexist. Understanding this representational structure allows us to reflect on the challenges of professional training and the construction of nurses’ identity, as well as on the need to deconstruct stigmas that still permeate nursing.

The idealized representation and perception of professional reality point to the need for training that goes beyond the technical dimension. It is essential that the educational process offers reflective and critical support, encouraging students to understand the historical, social, political, and ethical aspects that shape nurses’ identity. Valuing the technical and scientific dimension of care, without losing sight of its humanized nature, is a fundamental condition for the consolidation of a safe, qualified, and transformative practice.

This study highlights the importance of incorporating spaces for discussion into the undergraduate nursing curriculum that promote the strengthening of professional identity, the valuing of nurse autonomy, and a broader understanding of nursing’s social role.

Students’ social representation not only reflects perceptions about what it means to be a nurse, but also acts as a mediator in the construction of meanings that shape, sustain, and challenge the training process and professional practice, thus revealing itself as a powerful analytical tool for rethinking the interface between training, identity, and professional recognition in the field of nursing.

## Data Availability

The research data are available only upon request.
