# Supplementary material for: Social Perceptions of Nurses for Nursing Students
Source: Rev Bras Enferm. 2026 Jul 24;79:e20250336. doi: 10.1590/0034-7167-2025-0336 (PMC13403635; doi:10.1590/0034-7167-2025-0336)
Supplement: Translation

# Translation: A Representação Social sobre o Enfermeiro para Estudantes de Enfermagem

**DOI:** 10.1590/0034-7167-2025-0336pt

**Authors:** Stéfany Araújo Fraga, Felipe Kaezer dos Santos, Luiza Mara Correia, Antonio Marcos Tosoli Gomes, Denize Cristina de Oliveira, Livia Fajin de Mello, Frances Valéria Costa e Silva, Helena Ferraz Gomes

**Affiliations:** I Universidade do Estado do Rio de Janeiro. Rio de Janeiro, Rio de Janeiro, Brasil

**Keywords:** Enfermagem, Representação Social, Enfermeiros, Estudantes de Enfermagem, Cuidados de Enfermagem.

## Abstract

**Objetivos:**

analisar a representação social sobre o enfermeiro entre estudantes de enfermagem.

**Métodos:**

estudo qualitativo, fundamentado na abordagem estrutural da Teoria das Representações Sociais. Participaram 114 estudantes de enfermagem de uma universidade pública do Rio de Janeiro. A coleta de dados utilizou a técnica de evocação livre de palavras, analisadas com o apoio do *software* EVOC^®^.

**Resultados:**

o provável núcleo central da representação foi composto pelos termos “cuidado”, “empatia” e “saúde”. Os elementos periféricos de destaque foram “amor”, “conhecimento”, “desvalorização” e “cansaço”. A zona de contraste apresentou os termos “responsabilidade”, “assistência” e “dedicação”, reforçando os termos do núcleo central.

**Considerações Finais:**

a representação social do enfermeiro é estruturada fortemente em torno do cuidado, ligada à empatia, ao conhecimento e à responsabilidade ética. Embora permeada por afeto e compromisso, emergiram imagens relacionadas à desvalorização e sobrecarga, revelando tensões entre a formação idealizada e a realidade da profissão.

## INTRODUÇÃO

O conceito de cuidar é um atributo intrínseco ao humano e essencial para a manutenção da humanidade, sendo considerado a essência da profissão de enfermagem. Assim, desde o surgimento da enfermagem profissional, o cuidado passou por transformações muito significativas, desde as primeiras abordagens ainda bastante primitivas até a consolidação das práticas por meio de evidências^(1)^.

Com a evolução dos cuidados, a enfermagem também progrediu, especialmente a partir do século XIX, quando Florence Nightingale cuidou dos militares feridos na Guerra da Crimeia (1853-1856), aplicando conceitos básicos como ventilação, higiene e nutrição dos feridos em combate^(2)^.

É possível pressupor que, àquela época, as ações propostas por Florence tenham sido instauradas a partir da enfermagem concebida por ela, destoando da compreensão sobre o trabalho da enfermagem vigente até aquele momento, e movidas por uma concepção singular e inovadora do que significava ser enfermeira. Para além das atividades assistenciais, tratava-se de aplicar raciocínio lógico, observação minuciosa, organização do ambiente e responsabilidade ética com base em evidências. A partir do conhecimento científico e de práticas próprias, ela propôs, então, um modelo de cuidar voltado à promoção da saúde e prevenção de doenças, e não apenas à cura^(3)^.

No Brasil, apenas no século XX, a enfermagem passou a ser reconhecida como ciência, a partir da consolidação dos modelos teóricos de cuidado. Entre eles, destaca-se o modelo proposto por Wanda de Aguiar Horta voltado às necessidades humanas básicas e ao cuidado integral ao indivíduo^(4)^. Ao longo dos anos, surgiram diversas concepções teóricas de cuidar, contribuindo para a consolidação da ciência da enfermagem a partir do ser humano, do ambiente e da ação profissional^(5)^.

Em meio aos diversos avanços, atualmente existe um desafio considerável a ser superado relacionado ao reconhecimento social da categoria. Para tanto, existem entraves expressivos a serem superados, tais como a precarização do trabalho, a desvalorização decorrente das questões de gênero, a jornada exaustiva de trabalho e a baixa remuneração^(6)^. Transpor esses desafios é tarefa crucial para assegurar que a enfermagem siga exercendo seu papel transformador na área da saúde, fortalecendo as políticas públicas e os espaços acadêmicos de formação e pesquisa.

Há que se considerar que a atuação e o desempenho profissional do enfermeiro estão relacionados à representação que o indivíduo tem acerca de sua categoria profissional. Assim como ocorreu com Florence Nightingale, para ocupar uma determinada posição perante a sociedade e o mundo do trabalho, é preciso que o profissional compreenda a sua inserção, suas atribuições, competências e potencialidades^(7)^.

Neste sentido, o processo formativo assume importância central, pois, ao longo da graduação, o estudante de enfermagem avança em competências e na consolidação de sua identidade profissional^(8)^. Portanto, conhecer a representação que o estudante tem acerca da profissão pode fornecer subsídios para a reflexão acerca do reconhecimento da categoria e também da influência das experiências decorrentes do processo formativo sobre essa imagem.

A representação social é compreendida de forma individual e social simultaneamente. Sendo individual, engloba o sujeito, referindo-se ao seu pensamento. Sendo social, está inserida em um grupo. Ao longo da trajetória de vida, o pensar e o representar podem adquirir novas formas e possibilidades^(9)^. De acordo com Serge Moscovici, a representação social consiste no agrupamento de imagens e linguagem, com enfoque nas situações construídas pelas interações sociais, realizando a associação da compatibilidade do pensamento dos grupos e dos indivíduos. Logo, as representações contribuem para a construção do que é gerado no exterior de acordo com a relação que os grupos e os indivíduos constroem com os objetos^(10)^.

A representação estereotipada de uma profissão gera impacto significativo na percepção do trabalho da categoria. Historicamente, a imagem do enfermeiro esteve vinculada à representação social baseada em valores religiosos e na caridade. Embora essa percepção tenha evoluído ao longo do tempo, ainda não alcança o reconhecimento pleno da profissão^(11)^. Neste sentido, torna-se importante identificar a representação social do enfermeiro.

Atualmente, a rápida circulação de informações e a forte influência dos meios de comunicação impactam significativamente a construção social da enfermagem. A mídia, ao disseminar estereótipos que retratam o enfermeiro como um profissional sem autonomia, subordinado e com conhecimento limitado, reforça concepções equivocadas que prejudicam a sua valorização^(12)^.

Estudo realizado em 2018, com cerca de 60 estudantes de enfermagem, mostrou que as representações sociais dos estudantes sobre o enfermeiro ainda são marcadas por concepções tradicionais, muitas vezes associadas à figura do cuidador altruísta e à prática assistencial voltada à caridade e execução de tarefas. Essas percepções influenciam a construção da identidade profissional, sob o risco de comprometer a valorização da inserção social do enfermeiro, sua autonomia e seu reconhecimento^(13)^.

Em outro estudo, estudantes de enfermagem relataram frustração ao confrontar o ideal do perfil profissional construído na academia com a realidade prática, marcada por profissionais frequentemente exaustos e sobrecarregados em tarefas múltiplas. Ademais, destacou-se a imposição de um planejamento de cuidados centrado em intervenções médicas, gerando conflitos e incertezas acerca da identidade e do significado do exercício profissional do enfermeiro na prática cotidiana^(14)^.

Assim, é preciso considerar que a representação social do enfermeiro constitui um elemento fundamental para compreender como a profissão é percebida pelos próprios profissionais em formação. Tais representações influenciam diretamente a construção da identidade profissional, bem como a valorização social e simbólica da enfermagem. Infelizmente, é comum observar a reprodução de estereótipos que associam o enfermeiro à figura de auxiliar ou subordinado ao médico, reduzindo a complexidade e a autonomia que caracterizam o exercício profissional da enfermagem^(15)^.

Estudar a representação social do enfermeiro entre estudantes de enfermagem é, portanto, relevante para identificar como essas percepções se formam e são reproduzidas ao longo da formação acadêmica. Isso se torna ainda mais necessário diante de um contexto marcado pelo excesso de trabalho, múltiplas atribuições e falta de reconhecimento, fatores que desmotivam o exercício da profissão, impactando diretamente a qualidade do cuidado ofertado^(16)^.

A relevância desta investigação está em compreender como a representação do enfermeiro se forma e se transforma durante a trajetória acadêmica, influenciando a identidade profissional. Esse entendimento evidencia a enfermagem como profissão em constante desenvolvimento e ressignificação, reforçando a importância de promover reflexões que valorizem a categoria, desconstruam estigmas e reafirmem sua essencialidade no cuidado em saúde.

## OBJETIVOS

Analisar a representação social sobre o enfermeiro para estudantes de graduação em enfermagem.

## MÉTODOS

### Aspectos éticos

O estudo seguiu a Resolução CNS n^o^ 466/2012^(17)^, que define diretrizes éticas para pesquisas com seres humanos, assegurando direitos, segurança e bem-estar dos participantes. Após autorização institucional, foi registrado na Plataforma Brasil e aprovado pelo Comitê de Ética em Pesquisa (Parecer nº 6.913.915). A inclusão dos participantes ocorreu mediante concordância com o Termo de Consentimento Livre e Esclarecido, disponibilizado virtualmente conforme a Carta Circular nº 1/2021-CONEP/SECNS/MS^(18)^. O documento, em formato digital, permitiu leitura integral, com registro eletrônico da aceitação e subsequente envio de uma cópia ao participante.

### Referencial teórico-metodológico

Trata-se de estudo fundamentado no referencial teórico-metodológico da Teoria das Representações Sociais (TRS). Segundo Serge Moscovici, as representações sociais são fenômenos complexos que atuam diretamente nos grupos, sendo construídas por meio da interação social^(10)^. Para o autor, há uma relação direta entre o individual e o coletivo. Assim, a noção de representação permite compreender esse elo e como os significados construídos podem ser compartilhados^(19,20)^.

A fim de atingirmos o objetivo proposto para este estudo, foi utilizada a abordagem estrutural da TRS, que possui como elemento marcante a teoria do núcleo central, desenvolvida por Jean-Claude Abric em 1976^(21)^. Na abordagem estrutural, a representação social é compreendida como uma estrutura organizada e atravessada por diferentes dimensões. Nessa perspectiva, apenas identificar o conteúdo da representação não é suficiente; é necessário compreender também a forma como esse conteúdo se estrutura e se articula internamente^(22)^.

Portanto, a abordagem estrutural considera a representação como um conjunto organizado e estruturado de informações, crenças, opiniões e atitudes, constituindo um sistema sociocognitivo particular composto de dois subsistemas: um sistema central (ou núcleo central) e um sistema periférico^(23)^. A abordagem estrutural se vale da análise prototípica, que consiste na organização de palavras livremente evocadas a partir da menção de um termo indutor, de acordo com a frequência e a ordem média de evocação (OME), e dispostas em um quadro com quatro quadrantes. Essa composição, por sua vez, apresenta os termos evocados em agrupamentos que revelam o provável núcleo central da representação e seus componentes periféricos^(24)^.

A escolha da abordagem estrutural se deu por sua ampla utilização e sua aplicabilidade, permitindo dialogar construtivamente com variadas áreas do conhecimento^(25)^. Cabe mencionar que a concepção desta abordagem valoriza uma orientação metodológica experimental, enfatizando, assim, a estruturação dos conteúdos cognitivos das representações^(26)^. Por conseguinte, a aplicação dessa abordagem nos permite ampliar a compreensão acerca de um objeto de representação - o enfermeiro - para um grupo social: estudantes de graduação em enfermagem.

### Tipo de estudo

Trata-se de estudo com abordagem qualitativa e natureza descritiva, estruturado com base nos princípios metodológicos do *checklist COnsolidated criteria for REporting Qualitative research*, que oferece diretrizes padronizadas para a apresentação de pesquisas qualitativas, assegurando maior transparência, consistência e rigor na descrição dos procedimentos metodológicos.

### Procedimentos metodológicos

A coleta de dados foi realizada no período julho de 2024 e março de 2025, com a utilização de um formulário eletrônico, organizado em duas seções: a primeira é destinada à construção da caracterização sociodemográfica do grupo estudado; e a segunda é voltada à coleta da evocação livre de palavras. Foi solicitado que os participantes registrassem até cinco palavras para o termo indutor “enfermeiro”. A maior parte dos estudos que utilizam análise prototípica geralmente solicita a evocação de três a cinco termos por participante, sendo que esta técnica também pode ser utilizada com número diferente de respostas e até mesmo sem restrição de quantidade^(25)^.

### Cenário do estudo

O cenário do estudo foi a faculdade de enfermagem de uma universidade pública do estado do Rio de Janeiro, que conta com 320 estudantes, distribuídos em nove períodos acadêmicos. Foram incluídos estudantes de enfermagem com matrícula ativa na universidade, independentemente da orientação sexual. Foram excluídos os participantes com menos de 18 anos ou com formação prévia, em qualquer nível, na área da saúde. Essa opção se deu como forma de minimizar a influência de experiências prévias no contexto da assistência à saúde sobre a produção de dados.

### Fonte de dados

Foram incluídos estudantes de duas turmas do primeiro período e duas do nono (e último) período acadêmico. Assim, tivemos participantes de diferentes etapas do curso, representando o início e o término da formação. Dessa forma, buscou-se assegurar a heterogeneidade na amostra, permitindo capturar possíveis variações nas representações sociais ao longo do percurso acadêmico. O número de participantes foi definido pelo recorte das turmas selecionadas, com adesão voluntária dos estudantes, assegurando um tamanho de amostra adequado para a identificação do núcleo central e dos elementos periféricos da representação.

No total, 130 estudantes responderam ao instrumento de coleta de dados, o que equivale a 40,6% do corpo discente. A seleção dos participantes ocorreu por conveniência, estratégia amplamente utilizada em pesquisas qualitativas^(27)^, por facilitar o acesso ao grupo estudado e, neste caso, permitir a inclusão de estudantes em diferentes estágios do curso.

### Coleta, organização e análise dos dados

A coleta de dados foi realizada pela autora principal do estudo, que, à época, atuava como interna de enfermagem na instituição onde a pesquisa foi conduzida e com a qual mantinha vínculo direto. Para a execução da coleta de dados, a pesquisadora recebeu treinamento específico do professor orientador, baseado em leitura e discussão.

Os participantes foram abordados em sala de aula, com explicação detalhada sobre os objetivos e procedimentos da pesquisa, e convidados a participar de forma voluntária. A coleta de dados ocorreu por meio de formulário digital, de forma coletiva, mas individualizada e sem comunicação entre os participantes. Essa estratégia permitiu padronizar a aplicação do instrumento e garantir que todos compreendessem corretamente as instruções. Esse momento ocorreu apenas com a pesquisadora e os participantes, sem professores ou outros estudantes.

O conjunto dos dados produzidos pelos participantes foi organizado em uma planilha eletrônica e padronizados em um dicionário de evocações. Foram registrados, em média, quatro termos por participante, totalizando 561 evocações, consolidadas em 86 termos padronizados. Em seguida, o *corpus* de análise foi submetido ao *software* EVOC^®^ (versão 2005), que distribuiu as palavras nos quatro quadrantes do quadro de quatro casas de acordo com a frequência e a OME.

De forma complementar, foi realizada também a análise de similitude, uma técnica que estuda as distâncias entre os diversos elementos. As forças de ligação dos termos são apresentadas em forma de árvore, mostrando as distâncias entre eles. Os elementos próximos ou conectados a muitos outros tendem a ser considerados centrais. Por sua vez aqueles mais distantes, com poucas ligações e com força de ligação baixa tendem a ser considerados periféricos^(21)^.

Dessa forma, a árvore máxima de similitude (Figura 1) foi obtida a partir da determinação de coocorrência entre dois termos evocados: quanto maior a frequência de coocorrência, maior a força de ligação. Este índice foi calculado pela proporção entre o número de coocorrências de dois termos no *corpus* de análise e o número de participantes. A construção da árvore máxima de similitude foi realizada pelos autores deste estudo, priorizando as conexões com maior força de ligação.

**Figura 1:** Árvore máxima de similitude (representação social do enfermeiro para estudantes de enfermagem), Rio de Janeiro, Rio de Janeiro, Brasil, 2025

## RESULTADOS

Na Tabela 1, apresentamos os dados sociodemográficos dos participantes da pesquisa para a compreensão do perfil do grupo estudado. Os 114 participantes são, em sua maioria, do sexo feminino, totalizando 94 participantes (82,5%). As faixas etárias de maior predominância foram de 18 a 20 anos, com 41 participantes (36%), e de 21 a 23 anos, com 37 participantes (32,5%). Entre os participantes, 64 se declararam brancos (56,1%), e 30 participantes (26,3%) se declararam pretos.

**Tabela 1:** Caracterização sociodemográfica dos participantes, Rio de Janeiro, Rio de Janeiro, Brasil, 2025

Variáveis	Estudantes de enfermagem									
	Homem	%	Mulher	%	Não inf.	%	H. Trans	%	TOTAL	%
18 a 20 anos	6	5,3	33	28,9	2	1,8	0	0,0	41	36,0
21 a 23 anos	4	3,5	32	28,1	1	0,9	0	0,0	37	32,5
24 a 26 anos	4	3,5	22	19,3	0	0,0	1	0,9	27	23,7
27 a 29 anos	2	1,8	2	1,8	0	0,0	0	0,0	4	3,5
30 ou mais anos	0	0,0	5	4,4	0	0,0	0	0,0	5	4,4
Branca	6	5,3	57	50,0	1	0,9	0	0,0	64	56,1
Preta	6	5,3	23	20,2	1	0,9	0	0,0	30	26,3
Parda	4	3,5	14	12,3	1	0,9	1	0,9	20	17,5
TOTAL	16	14,0	94	82,5	3	2,6	1	0,9	114	100

*Não inf. - não informado; H. trans - homem transgênero.*

A análise do perfil dos 114 participantes põe em perspectiva os resultados da representação social do enfermeiro. A predominância feminina pode reforçar dimensões simbólicas associadas ao cuidado, historicamente valorizadas na enfermagem. A faixa etária indica o início da trajetória profissional do grupo, o que pode influenciar a priorização de elementos mais gerais ou idealizados da profissão. Além disso, a diversidade racial, com maioria branca e significativa presença de estudantes pretos, também pode afetar a percepção de experiências e valores profissionais, refletindo-se na organização central e periférica da representação social. Assim, gênero, idade e experiência de vida fornecem um contexto interpretativo relevante para a análise de dados.

O quadro de quatro casas (Quadro 1) é formado pelos termos evocados pelos participantes, considerando a frequência e a OME. No quadrante superior à esquerda, temos o núcleo central, composto por elementos estáveis. Ou seja, os cognemas deste quadrante são o cerne consistente da representação, menos afetados pelo ambiente externo ou pelas atividades dos indivíduos. Assim, compondo o provável núcleo central deste estudo, temos os elementos “cuidado”, “empatia” e “saúde”. O termo “cuidado” foi o mais frequente (102) e o mais rapidamente evocado (OME de 1,912).

**Quadro 1:** Quadro de quatro casas, Rio de Janeiro, Rio de Janeiro, Brasil, 2025

	OME < 3,0			OME < 3,0		
Freq. >= 22	Cuidado Empatia Saúde	Freq. 102 27 25	OME 1,912 2,963 2,480	Amor Conhecimento	Freq. 26 22	OME 3,231 3,318
Freq. >= 10 < 21	Responsabilidade Assistência Dedicação Acolhimento	Freq. 18 16 16 10	OME 2,444 2,313 2,875 2,700	Desvalorização Cansaço Gerência Militância Humanização Trabalho Atenção Auxílio	Freq. 19 16 15 14 12 12 11 11	OME 3,526 3,625 3,200 3,143 3,583 3,083 3,091 3,455

*OME - ordem média de evocação; Freq. - frequência.*

A seguir, temos os elementos periféricos da representação, que promovem a interface entre a realidade concreta e o sistema central, carregando a heterogeneidade e as aparentes contradições do grupo. A primeira periferia inclui os elementos mais importantes, os quais, em determinadas situações, podem se revelar centrais. A segunda periferia é composta por elementos mais voláteis e mutáveis, em função das experiências e influências mais imediatas sobre o grupo.

Assim, no quadrante superior à direita, temos a primeira periferia da representação, com elevada frequência de evocação, porém anunciada mais tardiamente. Neste quadrante, estão os termos “amor” e “conhecimento”, sendo o primeiro o mais evocado e o mais prontamente evocado. No quadrante inferior à direita, temos a segunda periferia, que inclui as palavras com baixa frequência de evocação e pronunciadas tardiamente. Destaca-se o termo “desvalorização”, com a maior frequência de evocação, embora o mais prontamente evocado tenha sido “trabalho” (OME de 3,083). Na zona de contraste, temos elementos evocados rapidamente, porém com baixa frequência de evocação. Nesta região, estão os termos “responsabilidade”, “assistência”, “dedicação” e “acolhimento”.

A partir do quatro de quatro casas, apresentamos a árvore máxima de similitude (Figura 1), evidenciando as conexões entre os elementos que compõem a representação do enfermeiro para o grupo estudado.

## DISCUSSÃO

O núcleo central é composto por elementos mais estáveis e consensuais da representação, sendo altamente compartilhados pelo grupo^(26)^. Neste estudo, o provável núcleo central apresenta três elementos com dimensões distintas, quais sejam “cuidado” (conceitual), “empatia” (afetivo-atitudinal positivo) e “saúde” (técnico-profissional). Dessa forma, o núcleo central assume as feições de um profissional tipicamente afetivo, marcado por imagens que parecem indicar uma representação idealizada. Isso pode se dar em função de o grupo estudado ser composto por jovens estudantes de enfermagem, sem experiência prévia na profissão ou na área da saúde, o que fica evidenciado pela ausência de elementos que expressam contexto, técnica e prática profissional.

O termo “cuidado” é um elemento conceitual multidimensional, amplo e consideravelmente abstrato que, por si só, não revela os componentes epistemológicos, ontológicos e metodológicos relacionados à representação social do enfermeiro^(28)^. Nesse sentido, para o grupo estudado, o cuidado possivelmente faz parte do processo de trabalho e é uma função primordial da enfermagem, abrangendo uma ampla variedade de atividades. Esse processo do cuidado (ou de cuidar) envolve outras questões associadas. Por se tratar de um conceito, pode incluir representações ligadas a questões psicológicas, físicas, técnicas e de procedimentos^(29)^.

Ao considerarmos o conjunto de elementos que compõem o provável núcleo central, observamos que o cuidado é acompanhado de um elemento afetivo positivo (empatia) e de um elemento associado ao trabalho técnico para produção da saúde. Isto parece sugerir que o cuidado, representado pelos participantes do estudo, pode conter em si elementos psicoafetivos e relacionais, mas também componentes técnicos e práticos, decorrente das próprias tarefas do cuidar. Nesse sentido, cabe reforçar que o cuidar em saúde envolve competências teóricas, técnicas e também científicas, pois é possível que os participantes representem a enfermagem como uma ciência que tem o cuidar como arte, estando presente em todas as etapas da vida^(30)^.

O termo “cuidado” apresenta a maior frequência e a menor OME, indicando que ele é o elemento organizador da representação do enfermeiro. Isso evidencia que o cuidado é o valor fundamental atribuído à profissão, sendo percebido como essencial. A presença de “empatia” e “saúde” neste quadrante reforça a imagem humanizada e voltada ao bem-estar do outro, revelando uma imagem positiva e fortemente associada ao perfil assistencial da profissão.

Estudo de representações sociais sobre “ser enfermeiro” para estudantes de enfermagem também apontou o cuidado como um elemento de centralidade, com semelhante comportamento no que se refere à frequência (f = 308) e à OME (1,960). Para os autores, apesar dos avanços técnico-científicos da profissão, há uma tendência natural dos profissionais de enfermagem de desempenhar suas funções de modo mais afetivo, associando as questões mais subjetivas da profissão e da convivência com o cliente durante a sua jornada de trabalho^(31)^.

Esse pensamento nos conduz à primeira periferia do presente estudo, que abriga elementos com alta frequência, mas com evocação mais tardia, indicando conteúdos mais adaptáveis ao contexto e à vivência prática dos sujeitos. Nele, o termo “amor”, de caráter afetivo-atitudinal positivo, foi o mais pronunciado, permanecendo neste quadrante apenas em função de sua OME.

A representação do amor parece remeter à origem da enfermagem, quando o ofício de cuidar era desempenhado por familiares ou por pessoas da caridade religiosa como forma de expurgar suas faltas. O elemento amor é reforçado pela imagem de “dedicação”, presente na zona de contraste. Ambos os termos indicam a persistente influência religiosa na representação do enfermeiro, firmada pelo contexto histórico de criação da profissão, reforçando a concepção de que este é o profissional cujo trabalho exige doação e entrega^(32)^. A idealização da profissão, permeada pelo afeto como instrumento de cuidar e como ato de filantropia, está associada à imagem de profissionais que o fazem por vocação, pelo dom de cuidar das pessoas, pois, a princípio, são bons e dedicados.

Os termos “amor” e “conhecimento”, que compõem a primeira periferia deste estudo, são os mesmos termos encontrados em estudo já mencionado anteriormente^(31)^. O conhecimento, em contraponto à ideia do cuidado por amor, pressupõe a fundamentação científica como princípio norteador da enfermagem. A apropriação do conhecimento, por sua vez, parece reforçar o termo “responsabilidade”, também presente na zona de contraste, na medida em que o trabalho responsável é feito com bases teóricas consistentes.

Assim, enquanto o “amor” indica o componente afetivo e vocacional frequentemente atribuído à enfermagem, o “conhecimento” remete à dimensão científica e técnica da profissão. A presença desses termos sugere uma sobreposição entre a idealização afetiva e a valorização técnica, mostrando que a profissão é considerada não apenas como cuidado permeado por compaixão, mas também como prática cientificamente fundamentada.

A segunda periferia, localizada no quadrante inferior direito, apresenta os elementos menos frequentes e evocados mais tardiamente, refletindo aspectos contextuais e experiências individuais. Nele, aparecem componentes que começam a fazer parte da vida profissional dos estudantes de enfermagem, como termos relacionados ao trabalho, mais concretamente, por exemplo, gerência, trabalho, atenção e auxílio, sendo que os dois últimos parecem reforçar o sentido de cuidado já mencionado.

Além disso, apresenta outros dois elementos de dimensão afetivo-atitudinais negativos em relação ao trabalho do enfermeiro, que são “desvalorização” e “cansaço”, expressando uma percepção mais crítica da realidade profissional, possivelmente influenciada por experiências de estágio ou pelo conhecimento de relatos sobre a precariedade do trabalho. A desvalorização do enfermeiro é uma discussão não somente para o grupo estudado, mas uma ideia geral da sociedade acerca da profissão. A ausência da informação sobre o papel do enfermeiro enfatiza e potencializa a desvalorização da profissão^(33)^.

Estudantes de enfermagem no Brasil frequentemente percebem que a profissão de enfermeiro é pouco valorizada, influenciados por relatos de condições adversas durante a formação, como carga horária excessiva, baixos salários e hierarquias rígidas. Outras condições como subalternidade em estágios clínicos, falta de autonomia e reconhecimento profissional limitado contribuem para sentimento de insegurança, baixa autoestima e dúvidas quanto à escolha da carreira. Esses sentimentos são reforçados pela observação de profissionais já formados que enfrentam jornadas exaustivas, escassa valorização institucional e baixa remuneração^(34)^.

Essa percepção tem impacto importante, uma vez que a desmotivação dos estudantes tende a aumentar a evasão e reduzir seu engajamento com a profissão. Além disso, a visão negativa sobre a valorização da carreira também pode limitar o desenvolvimento de competências de liderança e da identidade profissional, uma vez que os estudantes passam a perceber o exercício da enfermagem como uma trajetória de sacrifícios com pouco retorno^(35)^.

Diante das dificuldades que se apresentam para o grupo, a militância pode ser um elemento que caracteriza a busca por melhores condições de trabalho, reconhecimento e valorização profissional. A busca por melhorias de trabalho, muitas vezes, está associada ao movimento das categorias envolvidas, uma vez que, no Brasil, a força de trabalho em enfermagem é composta predominantemente por auxiliares e técnicos, formados em nível médio, que realizam tarefas assistenciais rotineiras, como aferição de sinais vitais, administração de medicamentos e cuidados de higiene, muitas vezes sem distinção clara entre suas atribuições^(36)^. Já os enfermeiros, com formação superior, assumem funções de planejamento, coordenação e decisão clínica. Essa divisão hierarquizada, por um lado, amplia o acesso aos cuidados de saúde, mas, por outro, evidencia disparidades salariais e de autonomia^(37)^, além de sobreposição de tarefas por parte de técnicos e auxiliares, resultando em fragilidade no exercício profissional^(36)^.

Essa realidade tem repercussões significativas. Os profissionais de nível médio frequentemente enfrentam precarização, insegurança no trabalho e sobrecarga emocional, enquanto a distinção entre as categorias pode gerar conflito e sofrimento moral dentro das equipes. Além disso, as desigualdades salariais são agravadas por questões étnico-raciais, com profissionais brancos recebendo, em média, mais do que os negros e pardos. Esses fatores comprometem a qualidade do cuidado, reduzem a visibilidade e o reconhecimento da enfermagem como profissão autônoma, demandando políticas que valorizem todas as categorias, e promovendo definição clara de atribuições e condições de trabalho justas para fortalecer o sistema de saúde^(38)^.

Os elementos situados na zona de contraste apresentam baixa frequência, embora sejam evocados precocemente. Tal configuração pode indicar a existência de um subgrupo de participantes que atribui elevada relevância a determinados elementos imagéticos ou, de modo alternativo, apenas reforçar os conteúdos já presentes nas demais zonas do quadro de quatro casas^(19,26)^, conforme examinamos a seguir.

Assim, consideramos que o termo “assistência” acompanha o sentido do cuidado e do trabalho, assim como o elemento “dedicação” pode se aproximar da empatia, do amor e da humanização em face do compromisso assumido com o outro. O termo “responsabilidade” aponta para uma representação que destaca a seriedade do trabalho do enfermeiro, ao passo que “assistência” e “acolhimento” reforçam o caráter relacional e afetivo do cuidado. Esses elementos podem indicar uma visão mais comprometida e reflexiva da profissão entre parte dos participantes.

Cabe destacar que o termo “acolhimento”, eminentemente polissêmico, carrega diferentes significados conforme o contexto em que é aplicado. Em sua acepção mais afetiva e relacional, acolher representa um ato de escuta empática, orientado pelo respeito à singularidade do outro, sendo essencial à prática humanizada da enfermagem. Nesse sentido, o acolhimento deve favorecer a construção de vínculos de confiança, constituindo a tecnologia leve do cuidado e garantindo o reconhecimento do sujeito como protagonista de seu cuidado^(39)^.

Por outro lado, o acolhimento assume uma dimensão estrutural nos serviços de saúde, especialmente na Atenção Primária e nos serviços de urgência, considerando-se a triagem ou classificação de risco. Nesse caso, a escuta inicial e a avaliação da urgência são organizadas por protocolos que garantem acesso e priorização técnica, o que confere um caráter operacional e institucional ao termo^(40)^.

Portanto, embora os elementos da zona de contraste possam, teoricamente, indicar a existência de subgrupos representacionais, no presente estudo, essa hipótese não se confirmou. A análise revelou que nenhum elemento apresentou distribuição diferenciada entre os subgrupos de estudantes, e todos os termos da zona de contraste estavam conectados aos elementos do núcleo central, sem formar agrupamentos diferenciados. Dessa forma, a zona de contraste parece reforçar elementos já presentes no núcleo central, confirmando sua função complementar na representação do enfermeiro.

A análise de similitude reforça a posição de centralidade do “cuidado”, elemento em torno do qual orbitam todos os demais. Esse dado parece sugerir que as representações do enfermeiro e do cuidado se sobrepõem, devido à proximidade entre o ser e o fazer na profissão de enfermagem. A árvore máxima de similitude nos permite retomar a discussão acerca dos significados do cuidado, uma vez que, considerando as forças de ligação entre os termos evocados, todos os demais elementos estão conectados a ele. Assim, em face da relevância na representação social do enfermeiro, todos os demais termos se referem ao “cuidado”.

O elemento “cuidado” reúne todas as maiores forças de ligação, permanecendo no centro da árvore de similitude, e os demais termos, como “empatia” e “saúde”, estão fortemente ligados a ele. Podemos identificar também que o termo “responsabilidade”, originalmente na zona de contraste, está bem mais próximo à centralidade se comparado com outros elementos periféricos. Esse dado também se aproxima de outro estudo realizado com estudantes de enfermagem^(31)^, que possui cuidado e responsabilidade como componentes do núcleo central.

### Limitações do estudo

Apesar da relevância dos achados, o estudo tem como limitação o número de participantes, o fato de ter sido realizado em uma única instituição de ensino superior e a não realização de outros testes de confirmação dos elementos centrais da representação. Desejamos ampliar o número de participantes e de instituições, bem como a possibilidade de também aprofundar, em estudos futuros, a representação social do cuidado, devido à importância que ele assumiu nesta pesquisa e como forma de compreender melhor a própria representação do enfermeiro, considerando a conexão entre ambos.

### Contribuições para a área da enfermagem, saúde ou políticas públicas

Ao final do estudo, os achados nos permitem refletir sobre a necessidade de reconsiderar as estratégias de formação profissional, de modo que os futuros enfermeiros se reconheçam e se valorizem pela relevância que a profissão possui e pela qualidade do próprio trabalho. Além disso, vislumbramos a necessidade de instituir políticas que promovam melhores condições de trabalho, oportunidades de carreira e suporte emocional durante a formação, fortalecendo a percepção de que a enfermagem é uma profissão essencial no âmbito da saúde digna e autônoma.

## CONSIDERAÇÕES FINAIS

Os achados deste estudo oferecem contribuições para o campo da formação do enfermeiro, sobretudo ao evidenciar como os estudantes constroem e compartilham representações sociais acerca da profissão durante a graduação.

Como síntese, temos que a representação social sobre enfermeiro se estrutura em torno do cuidado, ancorada em valores como empatia e conhecimento, a partir de uma dupla valorização: em primeiro lugar, o afeto e o compromisso; em seguida, a competência técnica e científica. No entanto, emergiram também representações negativas ou críticas ligadas à desvalorização e sobrecarga de trabalho, que revelam a tensão entre o ideal formativo e a realidade da profissão.

A centralidade do “cuidado” confirma que a essência do trabalho do enfermeiro é, desde cedo, internalizada como compromisso com o bem-estar do outro. A forma como o cuidado é representado - permeado por valores afetivos como empatia, amor e dedicação, e tensionado por elementos como desvalorização e cansaço - revela uma importante dualidade que deve ser discutida nos espaços formativos. Os termos “empatia” e “saúde” compõem uma imagem humanizada e orientada à integralidade, evidenciando o caráter relacional que permeia a identidade profissional do enfermeiro.

A presença de elementos como “amor” e “conhecimento” na primeira periferia demonstra a coexistência, respectivamente, de valores afetivos e técnicos, indicando uma representação que reconhece tanto a dimensão emocional quanto a científica da prática profissional. Por outro lado, o surgimento de termos como “desvalorização” e “cansaço” aponta para um conflito simbólico entre o ideal formativo e a realidade vivenciada ou observada no contexto prático, revelando os impactos da precarização do trabalho e da baixa valorização social da profissão.

Esses resultados refletem a existência de uma representação social marcada por desgastes, na qual convivem tanto a valorização simbólica do cuidar quanto as percepções críticas das condições de exercício profissional. Compreender essa estrutura representacional permite refletir sobre os desafios da formação profissional e da construção da identidade do enfermeiro, bem como sobre a necessidade de desconstruir estigmas que ainda permeiam a profissão.

A representação idealizada e a percepção sobre a realidade profissional apontam para a necessidade de que a formação que vá além da dimensão técnica. É imprescindível que o processo educativo ofereça suporte reflexivo e crítico, estimulando os estudantes a compreender os aspectos históricos, sociais, políticos e éticos que moldam a identidade do enfermeiro. Valorizar a dimensão técnica e científica do cuidado, sem perder de vista a sua natureza humanizada, é uma condição fundamental para a consolidação de uma prática segura, qualificada e transformadora.

Este estudo destaca a importância de incorporar, no currículo de graduação em enfermagem, espaços de discussão que favoreçam o fortalecimento da identidade profissional, a valorização da autonomia do enfermeiro e a compreensão ampliada do papel social da enfermagem.

A representação social dos estudantes não reflete apenas as percepções sobre o ser enfermeiro, mas opera como mediadora na construção de sentidos que moldam, sustentam e tensionam o processo formativo e o exercício profissional, revelando-se, assim, como instrumento analítico potente para repensar a interface entre formação, identidade e valorização profissional no campo da enfermagem.

## Data Availability

Os dados de pesquisa só estão disponíveis mediante solicitação.
